# A new distribution record of Arnold’s Gecko, *Pristurusminimus* (Arnold, 1977) (Squamata, Sphaerodactylidae), in Saudi Arabia

**DOI:** 10.3897/BDJ.11.e101647

**Published:** 2023-07-17

**Authors:** Mohammed Al Mutairi, Abdulaziz R Alqahtani, Zaffar Rais Mir, Riyaz Ahmad, Saad Alsubaie, Michael Smith

**Affiliations:** 1 National Centre for Wildlife, Riyadh, Saudi Arabia National Centre for Wildlife Riyadh Saudi Arabia; 2 University of Bisha, Bisha, Saudi Arabia University of Bisha Bisha Saudi Arabia

**Keywords:** new distribution record, protected area, Saudi Arabia, habitat, *Pristurusminimus*

## Abstract

Reptiles are considered an important element of Saudi Arabia’s wildlife to be conserved as a priority. However, the status and distribution of the Kingdom’s reptile fauna is not well understood, thus hindering the conservation initiatives. Better understanding of the taxonomy and distribution of the Kingdom’s reptile fauna is important for implementing effective conservation measures.

Here, we provide the new distribution record of *Pristurusminimus* (Arnold, 1977; common name, Arnold’s Gecko) from southern Saudi Arabia. The species was recorded from the Uruq Bani Ma'arid Protected Area (UBM) of southern Saudi Arabia. Four individuals were captured from different survey sites across the UBM and their species identification was confirmed through a mixture of physical and genetic examination. These results increase the number of species from the *Pristurus* genus to five for Saudi Arabia and improve the understanding of the Kingdom’s reptile fauna and its distribution.

## Introduction

The genus *Pristurus* RUPPELL 1835 (Squamata, Sphaerodactylidae), includes at least 20 species, most of which are found in Africa and the Arabian Peninsula (Papenfuss et al. 2009). Species in this genus are particularly known for the way they signal with body postures and tail movements (Castilla et al. 2014) and their diurnal and nocturnal habits. Compared to its congeners, *P.minimus* (Arnold 1977; common name, Arnold’s Gecko) is a small and slenderly-built lizard with a narrow head (Arnold 1980, Tamar et al. 2019). The species has been associated with hard sandy areas in the vicinity of shrubs and smaller plants (Arnold 1980) and areas of sparse vegetation, bare gravel, rock and/or sand (Carranza et al. 2018).

Up until now, *P.minimus* was known from Oman and the United Arab Emirates (refer to Fig.1 in Tamar et al. (2019)). Tamar et al. (2019) noted there was one record of the species from Saudi Arabia, refering to Arnold (1977). However, the detailed work of Arnold (1980) on reptiles of Arabia shows the distribution of *Pristurusminimus* in south-eastern Arabia and mentions the UAE and Oman only. Additionally, the recent study by Šmíd et al. (2021) does not list *P.minimus* in Saudi Arabia. Based upon their phylogenetic analysis, Papenfuss et al. (2009) listed *P.minimus* as sharing a clade with *P.carteri* (occurs in Oman, Saudi Arabia, UAE and Yemen), *P.crucifer* (occurs in Eritrea, Ethiopia, Kenya, Saudi Arabia and the Republic of Somalia) and *P.somalicus* (occurs in the Republic of Somaliland and Ethiopia). Tamar et al. (2019) split *P.minimus* into two species, *P.minimus* and *P.masirahensis*. *Pristurusmasirahensis* is endemic to Masirah Island (Tamar et al. 2019).

Here, we report on a range extension (≈ 850 km) and new record of *P.minimus* in the Uruq Bani Ma'arid Protected Area (UBM) of Saudi Arabia.

## Methodology

### Study Area

**Figure 1.**
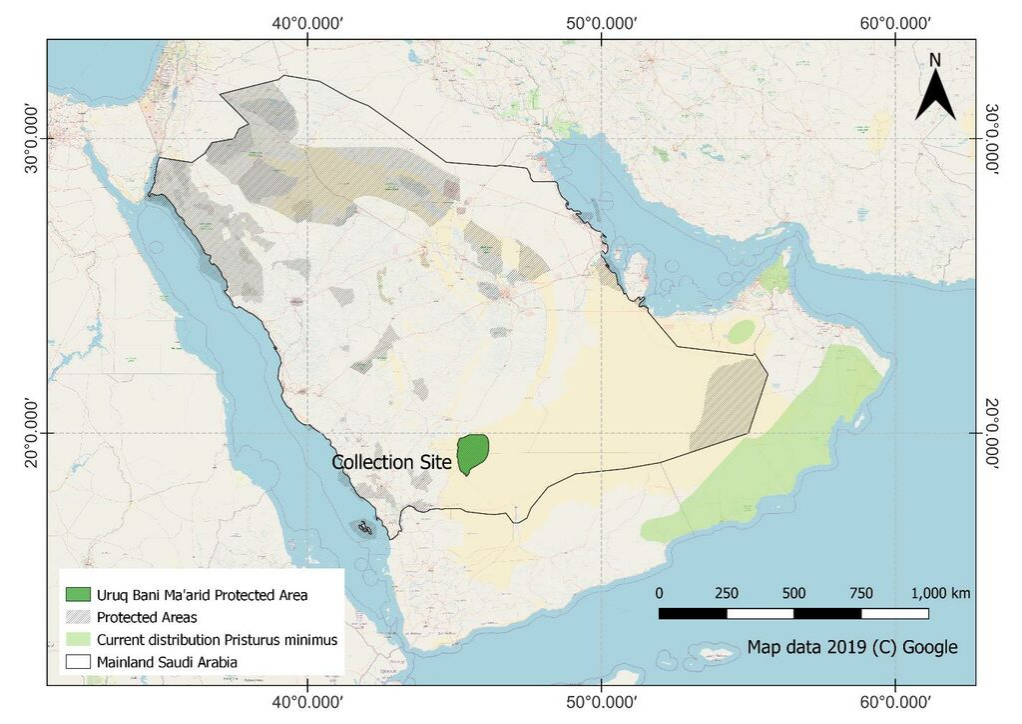
General location information for *Pristurusminimus*, including the location of the Uruq Bani Ma'arid Protected Area and the current distribution of *P.minimus* – provided by IUCN (2022).

The current study was conducted in the Urq Bani Ma’arid (UBM) Protected Area (Fig. 1). The UBM is situated in the western edge of Empty Quarter in Saudi Arabia (Islam et al. 2011). It has an area of around 12,787 km^2^, an elevational range from 720 to 940 m and a mean annual rainfall of around 47 mm (Aloufi et al. 2022). The UBM features rocky areas adjacent to longitudinal sand dunes (Aloufi et al. 2022) and a range of different habitats (e.g. vegetated wadis, plateaus, gravel plains and inter-dune corridors) (Islam et al. 2011). The UBM is known for the three ungulate species that occupy the area viz. Arabian Oryx, Sand Gazelle, Mountain Gazelle and the Spiny-tailed lizard (*Uromastyxaegyptia*; Wilms et al. (2012)). The UBM is also home to a rich carnivore assemblage including Rupell’s fox, sand cat and Honey Badger. It is also an ‘Important Plant Area’ and an ‘Important Bird Area’ (Hall et al. 2011, Aloufi et al. 2022).

### Field Surveys

**Figure 2.**
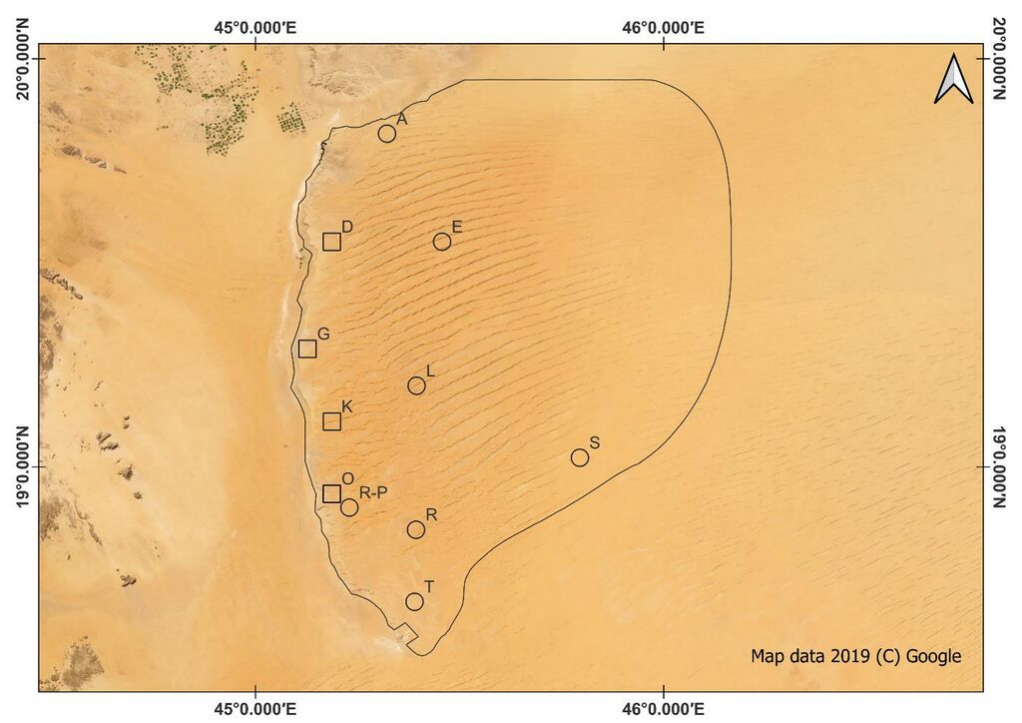
Sites where the *Pristurusminimus* was detected (square) and those surveyed, but *P.minimus* was not detected (round).

As part of monitoring the reptile diversity in Protected Areas of Saudi Arabia, repeated temporal and spatial surveys were conducted in UBM in years 2021 and 2022. Four surveys were conducted during different seasons and across different sites in UBM. The surveys were conducted both during day and night hours (6:00 am to 1:00 pm; 3:00 pm 11:30 pm) to document nocturnal as well as diurnal species. At a series of survey sites (Fig. 2), surveyors walked around searching for reptiles and, for every sighting, species, date, time, habitat and GPS coordinates were recorded. The individuals were photographed to aid in correct identification. The photographs were observed and compared with the photographs of *P.minimus* from Oman for ease of identification. The photographs were also sent to a leading expert on Geckos to validate the identification (Aaron Bauer, pers comm.).

#### Collection of Samples and Genetic Analysis

A genetic approach was used as a final confirmation of the species identification. Individuals were captured by hand for sample collection. Samples from tail were collected and preserved in alcohol vials for genetic examination. For one mitochondrial fragment of the gene encoding the ribosomal 12S rRNA, DNA was analysed after being extracted using the Qiagen DNeasy Blood and Tissue Kit (Catalogue no. 69506). DNA amplification was used on the DNA using 12S forward and reverse primers (12S; primers 12Sa and 12Sb; Kocher et al. (1989)) with 50˚C annealing temperature and 1.5 mM MgCl_2_. PCR amplifications were checked on agarose gel and the PCR products were sent to Macrogen Inc. in Korea for DNA sequencing. Using BLASTn criteria analysis, we compared our dataset of 388 bp with those from GenBank and the sequences were 98% identical with *P minimus*. Finch TV 1.4.0 was used to screen and analyse the sequences, which were aligned using ClustalW in Mega 6 using the default settings (Tamura et al. 2013). All sequences, apart from one sample from Saudi Arabia, were retrieved from GenBank under their accession numbers (given in the Results section). To estimate the sequence divergence for the whole dataset, genetic distances were calculated using Mega 6. Phylogenetic analyses were performed on separate analyses on the individual gene to determine the signal in the individual gene. The maximum parsimony (MP) and Neighbour-Joining (NJ) analyses were performed with Paup v.4 (Swofford and Sullivan 2009) with heuristic searches using step-wise addition, followed by tree bisection reconnection (TBR) branch swapping. In all alignments, gaps were treated as missing characters. Confidence within the nodes was evaluated using 1,000 bootstrap replicates (Felsenstein 2009) with random addition of taxa.

## Results

**Figure 3.**
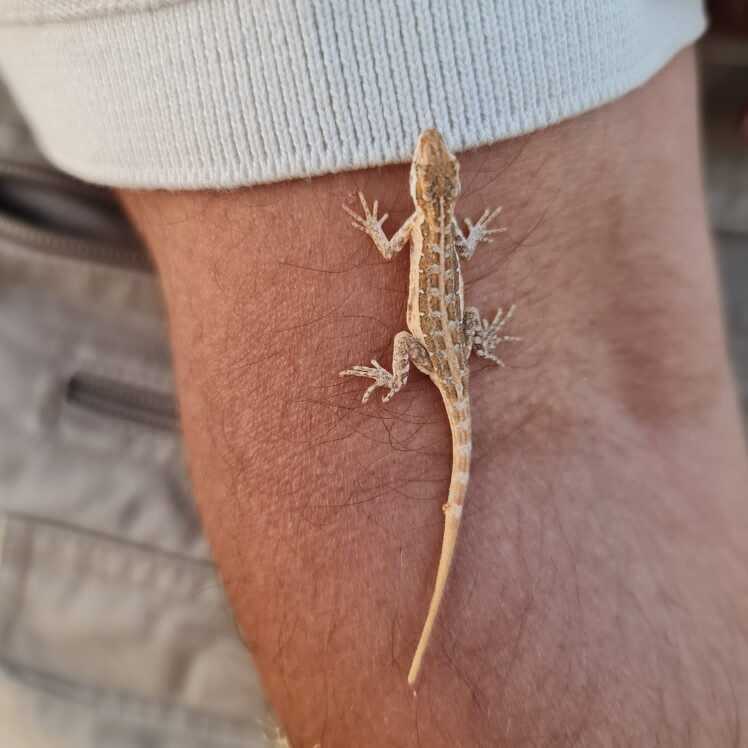
*Pristurusminimus* specimen photographed in Uruq Bani Ma'arid Protected Area.

Five *P.minimus* individuals were detected at four sites (Table 1, Figs 2, 3). Two individuals were collected at site ‘O’ (Fig. 2). The individuals were found in rocky habitats with sparse shrubby vegetation.

### Confirmation of Species identity

**Figure 4.**
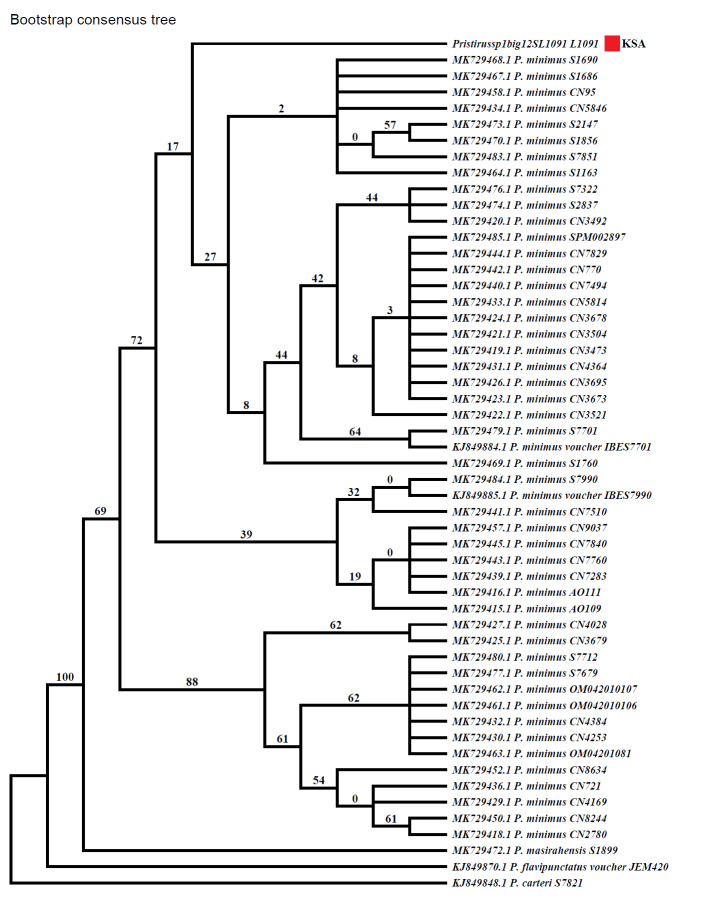
The maximum parsimony (MP) analysis result of *P.minimus* from Saudi Arabia.

**Figure 5.**
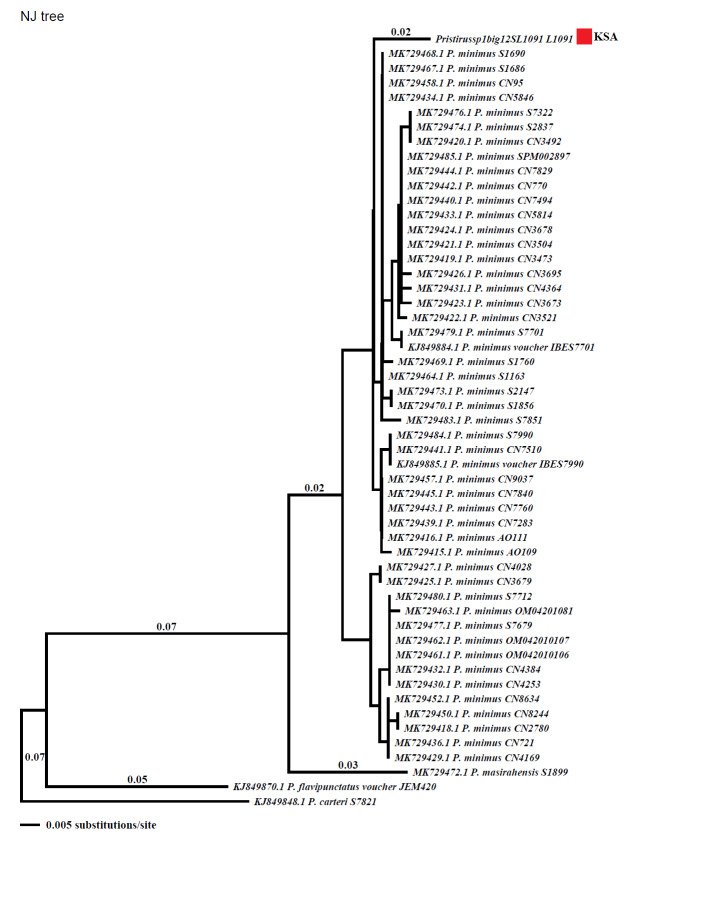
The Neighbour-Joining (NJ) analysis result for *P.minimus* from Saudi Arabia.

Photographs were used to provide initial confirmation of specific identity. The photographed individuals were identified as *P.minimus* on the basis of morphological characteristics as described by Wolfgang and Rolf (1990), Carranza et al. (2021), Burriel-Carranza et al. (2022). Species identification was then further confirmed with the help of an expert on Arabic geckos (Aaron Bauer, pers. comm). Genetic results using maximum parsimony (MP) and Neighbour-Joining (NJ) further validated the species identity by revealing Saudi Arabian *P.minimus* as a sister clade of Oman group (Figs 4, 5).

## Discussion

Wildlife research and conservation in Saudi Arabia have been taking place for some time although the focus was on large mammals mainly till recently. Quantifying the state of the reptile fauna of Saudi Arabia has received considerable focus of late (Al-Sadoon 1989, Al-Sadoon 2010, Al-Sadoon et al. 2017, Aloufi et al. 2019) resulting in the discovery of many unreported species across the country. Such discoveries have encouraged researchers to continue the exploration and documentation of the rich reptile fauna with morphological and genetic approaches of identification. However, vast sand deserts with a harsh environment and less diversity like UBM have not received much attention in terms of reptile studies. UBM has only been recently explored for reptiles (Cunningham 2010, Aloufi et al. 2022, Al Mutairi et al. 2023, in review) including our study, within the last three years. Up until this report, Sphaerodactylidae was represented by four species in Saudi Arabia, which can now be increased to five, with the inclusion of *P.minimus* from the UBM (Cunningham 2010, Aloufi et al. 2022, Al Mutairi et al. 2023, in review). With its position at the edge of Empty Quarter and escarpment, UBM provides a variety of habitats that can support a high diversity of faunal elements, including reptiles, warranting further exploration of this protected area and sites with similar levels of habitat heterogeneity (Al Mutairi et al. 2023, in review). The species has been earlier reported from southeast Arabia (Arnold 2009), UAE (Wolfgang and Rolf 1990) and Oman (Carranza et al. 2021, Burriel-Carranza et al. 2022). Our genetic evidence showed that there is an internal division within Arabian *P.minimus*. The genetic distance of Saudi Arabian species could result from the geographical isolation since the Empty Quarter could represent a barrier between the closest extension range of this species in the UAE and Oman.

Sphaerodactylidae are characterised by some of the smallest lizard species, which may contribute to their being more difficult species to detect during visually-based surveys. This further highlights the need to conduct repeated surveys in an area, preferably utilising robust design. Sites should be visited repeatedly in a short timeframe (i.e. within a few weeks of each other; secondary surveys) within sensible primary periods (e.g. annually, biennially or triennially during a period of time that species are most likely to be active; Kery and Royle (2020), Kéry and Schaub (2012)). With this new report, we believe that more people will be encouraged to focus on the exploration of UBM and other important areas for reptiles and, as such, we expect to see new records and range extensions.

## Conclusions

A new species, *P.minimus*, of the family Sphaerodactylidae has been recorded in Saudi Arabia for the first time. Five species from this genus are now known to occur in Saudi Arabia. Fortunately, the records were collected in a protected area, Uruq Bani Ma'arid Protected Area, providing some confidence that the species will be conserved.

## Figures and Tables

**Table 1.** Site and survey details for each individual *Pristurusminimus* detection. Site locations shown in Fig. 2.

**Site code**	**Elevation (m above sea level)**	**Survey time (AST)**	**Date**
D	889	17:30 to 22:00	06/04/2021
G	980	9:30 to 14:30	03/06/2021
K	942	9:30 to 14:30	01/03/2021
O	1021	9:30 to 14:30	06/03/2021
O	1021	9:30 to 14:30	02/06/2021
